# Recognition of depression and anxiety and their association with quality of life, hospitalization and mortality in primary care patients with heart failure – study protocol of a longitudinal observation study

**DOI:** 10.1186/1471-2296-14-180

**Published:** 2013-11-27

**Authors:** Marion Eisele, Eva Blozik, Stefan Störk, Jens-Martin Träder, Christoph Herrmann-Lingen, Martin Scherer

**Affiliations:** 1 Department of Primary Medical Care, University Medical Center Hamburg-Eppendorf, Martinistr. 52, Hamburg 20246, Germany; 2 Comprehensive Heart Failure Center, University of Würzburg, Straubmühlweg 2a, Würzburg 97078, Germany; 3 Department of Primary Medical Care, University of Luebeck, Ratzeburger Allee 160, Luebeck 23538, Germany; 4 Department of Psychosomatic Medicine and Psychotherapy, University of Göttingen Medical Center and German Center for Cardiovascular Research, von-Siebold-Str. 5, Göttingen 37075, Germany

**Keywords:** Heart failure, Depression, Anxiety, Quality of life, Prognosis, Observational study, Health care research, Mortality, Treatment, Hospitalization

## Abstract

**Background:**

International disease management guidelines recommend the regular assessment of depression and anxiety in heart failure patients. Currently there is little data on the effect of screening for depression and anxiety on the quality of life and the prognosis of heart failure (HF). We will investigate the association between the recognition of current depression/anxiety by the general practitioner (GP) and the quality of life and the patients’ prognosis.

**Methods/Design:**

In this multicenter, prospective, observational study 3,950 patients with HF are recruited by general practices in Germany. The patients fill out questionnaires at baseline and 12-month follow-up. At baseline the GPs are interviewed regarding the somatic and psychological comorbidities of their patients. During the follow-up assessment, data on hospitalization and mortality are provided by the general practice. Based on baseline data, the patients are allocated into three observation groups: HF patients with depression and/or anxiety recognized by their GP (P+/+), those with depression and/or anxiety not recognized (P+/−) and patients without depression and/or anxiety (P−/−). We will perform multivariate regression models to investigate the influence of the recognition of depression and/or anxiety on quality of life at 12 month follow-up, as well as its influences on the prognosis (hospital admission, mortality).

**Discussion:**

We will display the frequency of GP-acknowledged depression and anxiety and the frequency of installed therapeutic strategies. We will also describe the frequency of depression and anxiety missed by the GP and the resulting treatment gap. Effects of correctly acknowledged and missed depression/anxiety on outcome, also in comparison to the outcome of subjects without depression/anxiety will be addressed. In case results suggest a treatment gap of depression/anxiety in patients with HF, the results of this study will provide methodological advice for the efficient planning of further interventional research.

## Background

Heart failure (HF) is an increasingly prevalent condition with high mortality and morbidity rates [1]. Dyspnoea, oedema, fluid retention, pulmonary congestion and fatigue comprise common symptoms of HF. Although not necessarily jointly present within each individual patient, these symptoms exert substantial detrimental influences on functional status and quality of life [1-3].

### Psychosocial risk factors

Various studies established the important influence of psychosocial factors on the course and prognosis of HF patients [4,5]. Depression affects 20–40% of patients with HF [6] and is 4–5 times more common in HF than in the general population [7]. Depressive symptoms are associated with a 2–3 fold increased mortality risk and were shown to act independently of biological (classical) risk factors [8,9]. Symptoms of anxiety are reported for 38-70% of patients with congestive heart failure [10].

For this reason national and international HF guidelines recommend the regular assessment of depression [11-15] and anxiety [11,12,15] in heart failure patients. However, even though recent evidence suggests that patients screened positive for depression have an increased risk for hospitalization, emergency department visits and mortality [16,17], there is no data available on the transformation of these recommendations into routine care of HF patients.

Different aspects of psychosocial health (e.g. depression, anxiety, social support) are highly interrelated. It is still unclear which factor or component may independently affect the prognosis of HF patients [5]. We are aware of two randomised controlled trials investigating the effects of selective serotonin re-uptake inhibition on morbidity, mortality and mood in depressed patients with HF [18,19]. While the MOOD-HF study is still on-going [18], the SADHART-CHF trial concluded that the lack of effects of sertraline, compared to placebo treatment, may be due to the effect of nurse support within the placebo group, where depression scores improved significantly [19]. However, these trials focus on pharmacological interventions in a tertiary care setting. There are no interventional studies in primary care examining complex interventions such as screening for depression/anxiety (and subsequent treatment initiated by the general practitioner) in HF patients. There are also no studies that examined the effect of recognition and subsequent treatment of depression/anxiety initiated by the general practitioner (GP) once depression and or anxiety have been recognized. Therefore, it will be important to determine whether there is a need for interventional research targeting the effect of treating depression and anxiety on the prognosis of HF in patients with HF in primary care.

### Recognition of depression and anxiety

In Germany, the recognition rate of depression in the primary care sector has been reported at 74% [20]. Recognition rates among HF patients are likely to be lower because of the symptom overlap of non-specific HF symptoms and symptoms of depression. We are aware of one study investigating recognition rates of depression and anxiety in ambulatory HF patients. This study found that 42.5% of patients screened positive for depression and/or anxiety also had a diagnosis of depression or anxiety in their medical records [21]. However, that study almost exclusively included men (98.7%) and did not investigate the prognostic implication of recognition/treatment of depression/anxiety. Despite the purported clinical relevance of depression and anxiety, no intervention studies investigated the effects of depression and anxiety modification on the prognosis of HF patients in primary care settings. Further, the evidence base to support such trials is slim because so far there is no observational data underlining an association between recognition of depression and anxiety and the depression/anxiety-outcome [22], nor data underlining the cardiovascular outcome in primary care patients with HF [23-25].

As a result of these considerations, the aim of the study is to evaluate the state of psychosocial health care (recognized and unrecognized depression/anxiety and subsequent treatment) and its influence on health related quality of life and prognosis in primary care patients with HF.

Given that the prevalence rates of depression in HF patients vary due to differences in and the threshold levels of the assessment tools used, appropriate cut-off values for self-administered instruments measuring anxiety/ depression in HF patients will be derived in a sub-study.

## Methods/Design

The study is designed as a multicenter, prospective, observational study investigating 3,950 primary care patients with HF in Germany. The following research questions will be addressed:

● How many patients with HF suffer from depression and/or anxiety?

● How are the recognition rates of depression and/or anxiety by general practitioners (GPs)?

● How often are which measures taken by the GP when depression and/or anxiety are recognized?

● Is there an association between the presence of depression and/or anxiety and quality of life and HF prognosis (hospital admittance and mortality risk)?

● Is there an association between the recognition of present depression/anxiety by the GP and quality of life and HF prognosis (mortality, hospital admissions)?

● Which factors are associated with non-recognition and with failure to initiate adequate interventions (pharmacological or psychotherapeutical)?

● Is there an association between the presence and the recognition of depression/anxiety by the GP and health care aspects (e.g. frequency of contacts with the GP)?

### Recruitment

Recruitment takes place in two steps: First: primary care practices are recruited; and second: the participating GPs invite their patients to participate in the study. Two study centers, Hamburg and Würzburg, recruit general practices in four recruitment regions: Hamburg, Kiel, Lübeck (northern Germany) and Würzburg (southern Germany). The recruitment of the primary care practices began in April 2012 and will last until January 2014. All primary care practices in Hamburg, Kiel, Lübeck and Würzburg and within a 50 km radius of these cities, (to include urban and rural areas), receive a written invitation to participate in the study. If not responding to the invitation, study staff contacts each general practitioner by phone.

In a second step, participating GPs apply the following search criteria to identify eligible patients from the electronic records: age 18 and above, last consultation within the last six months, and any diagnosis of heart failure (ICD-Codes beginning with I50, I11.0, I13.0, I13.2) documented within the last 5 years. Out of all patients, who meet the mentioned criteria those who passed away since the last consultation or suffer from dementia are excluded (for a complete list of all inclusion and exclusion criteria see Table 1).

**Table 1:** Inclusion and exclusion criteria

	**Observation group 1 (P+/+)**	**Observation group 2 P(+/−)**	**Observation group 3 P(−/−)**
**Inclusion Criteria**			
Informed consent	✓	✓	✓
Aged 18 years and older	✓	✓	✓
Diagnosis of heart failure within the last 5 years, validated by the GP on basis of his medical records	✓	✓	✓
At least one contact with the participating GP within the last 6 month	✓	✓	✓
Depression and/or anxiety	✓	✓	
Recognition of existing depression and/or anxiety by the GP	✓		
**Exclusion Criteria**			
Diagnosis of dementia given by the GP	✓	✓	✓
Not a regular patient of the participating GP’s practice	✓	✓	✓

All eligible patients are invited to participate in the study through a letter from their GP. This letter includes all written information regarding the study, as well as a consent form. Beside a study description, the patient information covers procedures of the study, data collection, processing and storage, as well as possibilities for withdrawal of consent. A phone number for contacting the study staff with questions and for further information is provided with the information package. The informed consent form is already provided with a randomly assigned ID number.

In case of interest, the patient sends the signed, informed consent form to the recruiting study center via a prepaid and pre-addressed envelope. The first patient was included in July 2012; the recruitment of patients will last until January 2014.

### Study endpoints

The primary outcome of this longitudinal study is the quality of life after 12-months of follow-up. The secondary outcome is the prognosis of heart failure in terms of hospital admissions and mortality risk within the 12-month follow-up period. Group comparisons will contrast patients with depression and/or anxiety recognized by the GP (observation group 1: P(+/+)), patients with depression and/or anxiety not recognized by the GP (observation group 2: P(+/−)) and patients without depression and/or anxiety whose GPs did not diagnose any depression or anxiety (observation group 3: P(−/−)). For further information see Table 1.

### Data collection

Data is collected at the baseline assessment and at the follow-up assessment 12 months later. In the baseline assessment the patient receives a self-administered questionnaire by mail (see Table 2), as well as a free return envelope. Five days after the questionnaire is sent the patients receive a reminder by mail. Based on the information provided in the questionnaire, the patients are assigned to one of three observation groups. For more details see section *Recognition of depression and anxiety*. The GP of each patient assigned to one of the observation groups is interviewed by study staff in a telephone-interview. During the interview the GP has access to all patient data in his or her medical records. The questions regarding depression and anxiety are collected along with a comprehensive list of further comorbidities (see Table 2).

**Table 2:** Data collection

**BASELINE**	
**Patients: Self-administered questionnaires (by mail)**	
Depression	Patient Health Questionnaire 9-item Subscale Depression (PHQ-9) [26-28]/Hospital Anxiety and Depression Scale [29,30] – Subscale Depression (HADS-D)/PROMIS Depression Scale [31,32]
Anxiety	Patient Health Questionnaire 7-item Subscale Generalized Anxiety Disorder Assessment (GAD-7) [33-35]/Hospital Anxiety and Depression Scale [29,30] – subscale anxiety (HADS-A)/PROMIS Anxiety Scale [31,32]
Treatment of Anxiety and Depression	Medication intake/Utilization of psychotherapy or other therapies (e.g. music therapy, occupational therapy, relaxation training)
Social support	Lubben Social Network Scale (LSNS) [36]/ENRICHED Social Support Instrument (ESSI) [37]
Stress	Patient Health Questionnaire - Subscale Stress (PHQ-D) [26,38]
Compliance	Morisky Medication Adherence Scale (MMAS-8) [39-41]
Quality of life	EQ-5D-5 L [42]
Self-efficacy	General Self-efficacy Scale [43]
Lifestyle	Salt restriction: Do you pay attention to your diet?/Fluid intake: How much do you drink daily?/Weight control: How often do you weigh yourself?/Vaccination: Did you receive a flu shot within the last year? Have you been vaccinated against pneumonia?/Alcohol intake: At occasions you drink alcohol, how much alcohol do you consume on average?/Smoking status and cigarette consumption: If you smoke occasionally or regularly: How much do you consume on average?
Physical Activity/Capacity	How often do you engage in physical activity? Based on Verghese [44]/Self-rated physical capacity according to NYHA class
Socio-economic status	Household income per capita
Sociodemographic data	Gender/Age/Height/Weight/Type of insurance/Marital status/Status of living/ Nursing care/Employment status/ Education [45]/Migration status/ Citizenship
Contact to the GP	Last contact/Frequency of contacts within the last 6 month/Years of being patient of the actual GP
**GPs: Telephone-interview with insight in the medical record**	
Validation of heart-failure diagnosis	Does the patient have heart failure?/Severity level according to New York Heart Association (NYHA) class/Ejection fraction
Size of practice	Number of GPs in the practice/ Practice type (single practice/practice sharing/group practice)/Number of patients insured by the statutory health insurance per quarter/ Percentage of private patients
Familiarity with the patient	Are you the regular GP of the patient?/How well do you know the patient? (Scale 1 to 10)
Comorbidity	Charlson Comorbidity Index [46]/Which additional diseases does the patient have?
Depression/Anxiety	Does the patient have any symptomatology of depression/anxiety? If yes, please specify/Had the patient any symptomatology of depression/anxiety in the past?
Measures taken if depression/anxiety is recognized	What treatment have you prompted?
Treatment of heart failure	What difficulties have you encountered when treating the heart failure in this patient? Have there been any reasons to deviate from the recommended pharmacological treatment?
Sociodemographic data of the GP	Age/Gender/Years of practice/Speciality/Additional trainings
**12-MONTH FOLLOW-UP**	
**Patients: Self-administered questionnaire (by mail)**	
*All measures according to baseline; additional data:*	
Hospital admission within the last 12 month	Number of Hospital Admissions/Date of admission/Length of stay/Admission diagnosis/Emergency vs. pre-scheduled hospitalization
Change of GP	Did you change your GP within the last 12 month? If yes: How many months ago did you change your GP?
**GP: Information from medical records (provided by phone)**	
Survival	Mortality within the last 12 month/Date of death/Course of death
Hospital Admission	Number of Hospital Admissions/Date of Admission/Length of stay/Admission diagnosis/Emergency vs. pre-scheduled hospitalization/

Since diagnosis of heart failure will be obtained from the electronic patient records, we will double-check that the heart failure diagnosis is correct at the beginning of the telephone interview with the GP. After validating the diagnosis, the GP is asked to specify the severity level of heart failure according to New York Heart Association (NYHA) functional class. For all further information collected see Table 2.

At the 12-month follow-up assessment the patient receives a second self-administered questionnaire by mail, while data in the general practice will be extracted from the medical records of the practice and are provided by either the GP or practice staff over the phone. For further details regarding the contents of the data collection please refer to Table 2.

### Recognition of depression and anxiety

A sub-study is performed by the University of Göttingen Medical Center, which is not involved in the main study. The aim of this sub-study is to identify appropriate cut-points and combinations of established self-rating scales for depression and anxiety in primary care patients with heart failure. For this purpose, a subsample of 190 patients with symptomatic heart failure completes the Hospital Anxiety and Depression Scale (HADS) [29,30], Patient Health Questionnaire 9-item Subscale Depression (PHQ-9) [26-28] and Patient Health Questionnaire 7-item Subscale Generalized Anxiety Disorder Assessment (GAD-7) [33-35], as well as selected items from PROMIS Depression and Anxiety scales [31,32], and undergoes the Structured Clinical Interview for DSM-IV (SCID) [47]. The SCID is a structured one-hour face-to-face diagnostic interview by a trained psychologist with a patient. It is the reference standard for the diagnosis of depression and anxiety disorders in research projects [48]. In this study SCID interviews are performed by psychologists (blind to patients’ self-ratings), to identify the relative value and optimal clinical cut-points for the identification of primary care patients with HF and comorbid depression or anxiety disorders. Different cut-points on the self-rated PHQ-9, GAD-7, and HADS are compared by Receiver Operating Characteristics for the detection of different classes of SCID-based diagnoses. If appropriate, exploratory analyses are attempted to optimise case finding algorithms based on combinations of the self-rating scales or their individual items. Previous research has shown that such analyses are feasible with the designated sample size for the sub-study [49,50].

In the main study, the case finding algorithm (identified in the SCID sub-study) is applied on the information given in the baseline questionnaire. Therefore, the questionnaire includes the same self-administered instruments as used in the sub-study (HADS, PHQ-9 and GAD-7, PROMIS Depression and Anxiety scales). Depending on the algorithm from the sub-study, the patients are classified as patients with depression and/or anxiety or patients without depression and/or anxiety. Due to the work load, we do not interview the GPs of 25% of the patients without depression and anxiety by phone. Therefore, for each practice 75% of all patients without depression and/or anxiety (group P-) are randomly selected for telephone interviews with the GP and assigned to the observation group 3 (P-).

**Figure 1:**
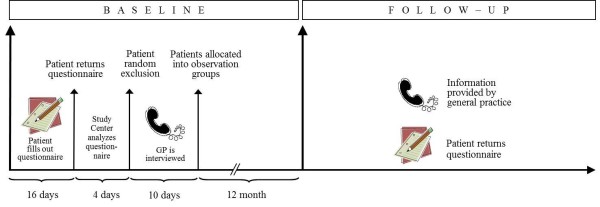
Baseline and follow-up assessment.

After baseline assessment, GP interviews will be available for each patient assigned to one of the three observation groups. For further detail see Figure 1.

The 75% randomly selected patients without depression and/or anxiety provides a higher number of patients in the observation group 3 (P−/−) than the power calculation suggests (see section *Sample size calculation*). The higher number is chosen for quality assurance. A group of patients without depression and anxiety which is too small would reveal the focus on depression and anxiety in this study. Therefore the proportion of patients with depression and/or anxiety and without depression/anxiety will be 1:3 in each interview.

Symptoms of anxiety and depression are addressed within a full list of somatic comorbidities, introduced by the somatic conditions of the Charlson Comorbidity Index [46] and followed by a question concerning any further comorbidity, which has not been addressed in the list before. The focus on depression and anxiety within this study is not revealed to the GPs. During the interview the GPs have full access to all patient data within their electronic records. The recognition of depression and/or anxiety is operationalized as the GP’s judgement in the telephone interview as to whether or not the patient displays symptoms of depression and/or anxiety. If the GP states that the patient displays symptoms, the GP is asked if he or she judges this as a clinical diagnosis or if he or she considers the symptoms not severe enough for a diagnosis.

In a next step, patient information from the questionnaire and GP judgment regarding depression and anxiety are matched and the patients are allocated to one of the following groups:

● Observation group 1 (P +/+): All patients with depression and/or anxiety whose depression and/or anxiety has been recognized by the GP

● Observation group 2 (P +/−): All patients with depression and/or anxiety whose depression and/or anxiety has not been recognized by the GP

● Observation group 3: P (−/−) Sample of patients without depression and anxiety who were randomly selected for telephone interview. In the interview the GPs did not diagnosed any depression or anxiety.

Patients without depression and anxiety whose GP nevertheless diagnosed depression or anxiety P(−/+) will be considered separately.

### Sample size calculation

Given a prevalence of psychosocial risk factors (depression, anxiety or both) in HF patients from general practices of 20% [6] and a recognition rate of 75% [20], we aim for a sample that provides us a minimum power of 80% to detect a minimum difference of at least any 5 points on the EQ-5D visual analogue scale (EQ-VAS) [42] with α = 0.05. Therefore, a sample of 672 participants with depression and/or anxiety is needed, of whom 504 have depression and/or anxiety recognized by their GP (P+/+) and 168 with depression and/or anxiety not recognized by their GP (P+/−).

**Figure 2:**
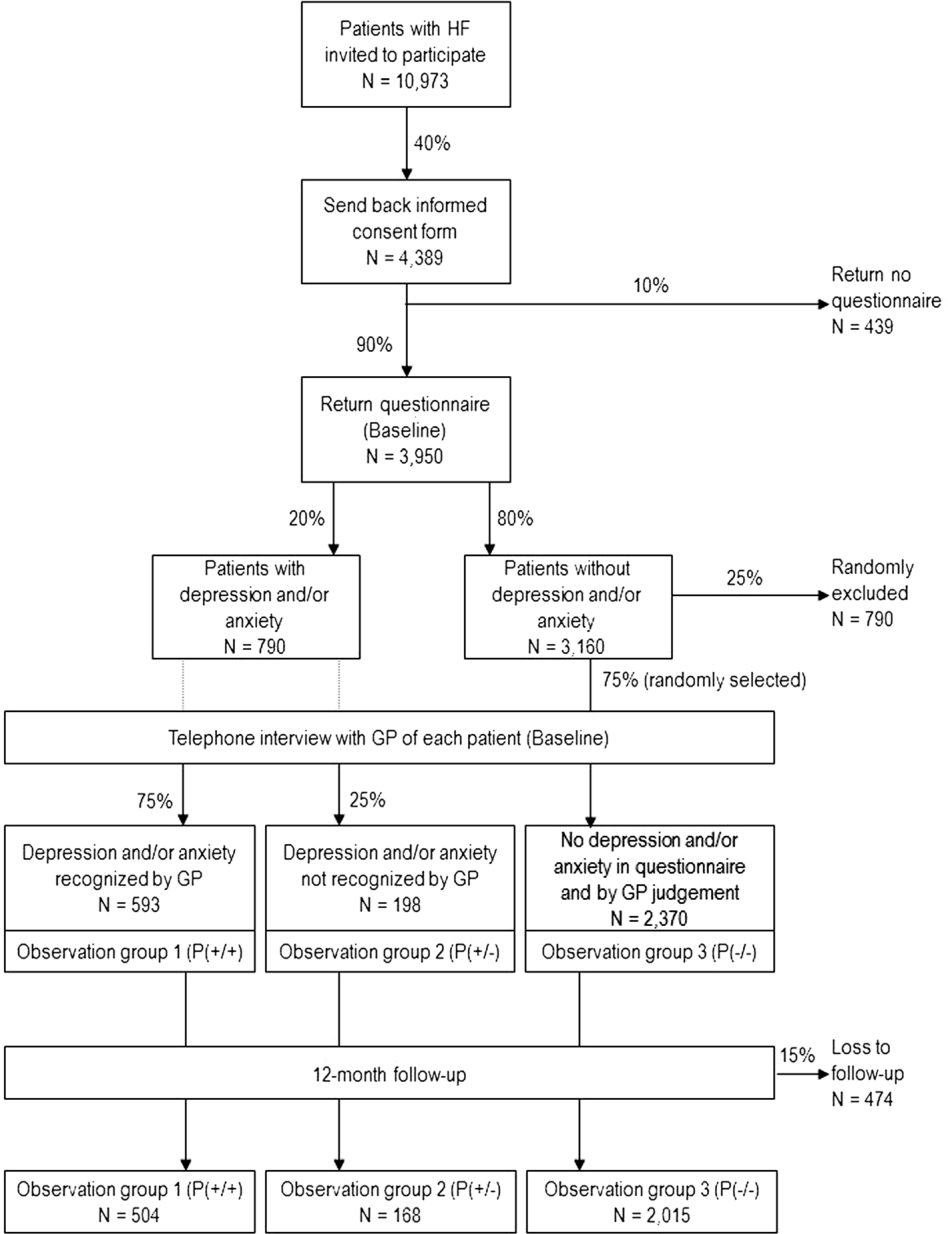
Sample size considerations.

Given an estimated participation rate of 40%, a return rate of the baseline questionnaire of 90% and a loss to follow-up rate of 15% a total of 10,973 patients with heart failure are needed to be invited into the study by their GP (see Figure 2).

For a comparison with heart failure patients without psychosocial risk factors, a random sample of N = 2,370 of those who completed the baseline questionnaire but were not identified to have depression and/or anxiety will be drawn (P−/−). Taking a loss to follow-up rate of 15% into account, a total of 2,015 patients with heart failure but without depression and/or anxiety at baseline will be included in the analyses. A minimum number of 118 patients at the follow-up assessment would already provide a power of 80% to detect differences between the groups (P+/+, P+/− and P−/−) of at least any 5 points on the EQ-5D visual analogue scale (EQ-VAS) [42] with α = 0.05.

### Quality assurance

The study is centrally coordinated by the study center Hamburg. All processes regarding the recruitment of GP practices and patients will be parallelized between both recruiting centers. To avoid selection bias, patients with heart failure are selected electronically from the medical records. We use validated instruments for the assessment of baseline and follow-up characteristics. All study staff conducting telephone interviews have been trained. All interviewers are blind regarding the group the patient has been allocated to.

For the data collection from GPs, depression and anxiety is offered in a full list of other potential comorbidities to minimise social desirability bias and to mask the focus on anxiety and depression. Additionally, the GP is interviewed regarding patients with and without depression and/or anxiety. The 75% of eligible patients in the observation group 3 exceed the number required by the power calculation and are suggested to be sufficient to detract from the focus on patients with depression and anxiety. Questionnaires are electronically imported into the database. All import data is double-checked before analysis. Analyses are performed anonymously.

### Data protection

All patients automatically receive a pseudonym which is printed on the consent form included in the study information they obtain. The data of the consent form is entered into a password protected database which is secured on an additional password protected USB stick stored in a lockable cabinet together with the consent forms.

All data assessed in the baseline and follow-up assessment is documented pseudonymously. The data is electronically entered in a local password-secured database. As soon as the data entry of the follow-up assessment is completed the allocation key will be deleted and all data will be anonymous.

### Description of risks

It is not expected that the participation in the study will expose the patients to any risks.

### Ethics approval

The study is conducted in compliance with the Declaration of Helsinki. The study protocol was approved by the local Ethics Committees (Medical Association of Hamburg, Approval No. PV3889; Ethics Committee of the Medical Faculty of the University of Würzburg, Approval No. 125/12, Ethics Committee at the University of Göttingen Medical Center, Approval No. 19/8/11).

### Data analysis

The prevalence of comorbidities in patients with HF and difficulties in the treatment of patients with HF will be analyzed by descriptive statistics. The prevalence of recognition/non-recognition of psychosocial risk factors in primary care HF patients and its impact on the study endpoints will be analysed. In a first step we will compare quality of life, hospitalisation and mortality after a 1-year follow-up period between HF patients with depression/anxiety (P+/+ and P+/−) and those without (P−/−).

In the second step we will compare quality of life, hospitalisation and mortality after a 1-year follow-up period between HF patients with depression and/or anxiety recognized by their GP (P+/+) and those with depression and/or anxiety not recognized (P+/−). We will perform multivariate regression models taking the confounding effects (e.g. comorbidity, sociodemographics, type of treatment for depression/anxiety and heart failure severity) into account.

In a third step we will compare several types of treatment for psychosocial risk factors and their potential effects on quality of life, hospitalisation and mortality after a 1-year follow-up period.

We will compare the information concerning the treatment of psychosocial risk factors gathered from patients with that gathered from GPs to evaluate potential differences (e.g. due to treatment initiated by other healthcare providers than the GP). We will re-run the analyses of the third step for information gathered from patients and for information gathered from GPs separately.

For binary outcomes (hospitalization, survival after 1 year), chi-squared tests will be used. For continuous outcomes (quality of life, survival time), t-tests or non-parametric rank tests will be used. Multivariate linear regression analyses will be performed for the total number of hospitalized days, Cox regression and Kaplan-Meier curves for time to hospitalization survival time. Multivariate linear regression will be used for quality of life.

## Discussion

This is the first study conducted to investigate the effect of the recognition of depression and anxiety and the subsequently initiated treatment by the GP on the quality of life and the prognosis of heart failure in HF patients in primary care. The results are expected to glean important insights into the care for HF patients in primary care.

### Expected implications for practice

We will be able to show how many heart failure patients in primary care suffer from depression and anxiety. We will display the frequency of GP-acknowledged depression/anxiety and the factors which may facilitate and exacerbate the recognition. Based on these results, patient characteristics which exacerbate recognition will be reported to GPs. Such knowledge will assist the GP in judging which HF patient may benefit from additional attention paid to symptoms of depression and anxiety. The frequency of installed therapeutic strategies and the impact of the different therapeutic approaches on quality of life and heart failure prognosis found in this study will be displayed. These results will be provided to GPs and will give a first orientation of which therapeutic strategies might be helpful for HF patients, not only to reduce depression, but also to improve their quality of life and prognosis of heart failure.

### Expected implications for health policy

Public health planners are in need of numbers such as frequency of depression and anxiety as well as frequency of different treatment options substantiating the decision process of resource allocation. For the health planners we will describe the frequency of depression and/or anxiety missed by GPs and the potential treatment gap.

Such a treatment gap with respect to psychosocial problems in HF patients is currently unproven but needs to be assumed if preliminary findings are transferred onto the general population. If so, more resources should be spent on investigating effective treatments.

For these investigations the differential effects of correctly acknowledged and missed depression and/or anxiety on outcome, also in comparison to the outcome of subjects without depression and/or anxiety, will help to better understand how health care for HF patients needs to be improved and which treatment might be effective. We will be able to present evidence to what extent the recognition of depression and/or anxiety through screening for depression and anxiety is mandatory for HF patients, as already recommended in clinical guidelines.

### Expected implications for research

The results of this study will help to generate hypotheses regarding effective treatment of depression and anxiety and the potential effects on the quality of life and prognosis in patients with HF. In a next step these hypotheses can be tested by intervention studies. The case-finding algorithm to identify HF patients with depression and anxiety obtained from the sub-study could be recommended for further observational and intervention studies in HF patients and is likely to reduce the huge variation of reported prevalence rates. In addition, this study will provide methodological support for efficient planning of further interventional research.

## Abbreviations

EQ-VAS: EQ-5D visual analogue scale; GP: general practitioner; GAD 7: Patient health questionnaire 7-item subscale generalised anxiety disorder assessment; HF: Heart failure; HADS: Hospital anxiety and depression scale; HASD-D: Hospital anxiety and depression scale – subscale Depression; HASD-A: Hospital anxiety and depression scale – subscale Anxiety; PHQ-9: Patient health questionnaire – 9 item depression subscale; PROMIS: Patient-reported outcomes measurement information system; SCID: Structured clinical interview for DSM-IV.

## Competing interests

CHL is receiving royalties from Hans Huber Publishers in Berne, Switzerland, for the German version of the Hospital Anxiety and Depression Scale. He received lecture honoraria from Servier, Pfizer and Berlin-Chemie as well as research funding from the statutory health-insurance organization KKH. All other authors declare that they have no competing interests.

## Authors’ contributions

ME drafted the manuscript, coordinates the study and contributed to the conception of the study. EB, SS and JMT contributed to the conception of the study. CHL contributed to the conception of the study and will conduct the sub-study. MS designed the study, drafted the grant proposal, contributes to the coordination of the study and is the principal investigator of the study. All authors critically revised the manuscript and agreed to the final version of the manuscript.

## Pre-publication history

The pre-publication history for this paper can be accessed here:

http://www.biomedcentral.com/1471-2296/14/180/prepub
